# WormBase 2024: status and transitioning to Alliance infrastructure

**DOI:** 10.1093/genetics/iyae050

**Published:** 2024-04-04

**Authors:** Paul W Sternberg, Kimberly Van Auken, Qinghua Wang, Adam Wright, Karen Yook, Magdalena Zarowiecki, Valerio Arnaboldi, Andrés Becerra, Stephanie Brown, Scott Cain, Juancarlos Chan, Wen J Chen, Jaehyoung Cho, Paul Davis, Stavros Diamantakis, Sarah Dyer, Dionysis Grigoriadis, Christian A Grove, Todd Harris, Kevin Howe, Ranjana Kishore, Raymond Lee, Ian Longden, Manuel Luypaert, Hans-Michael Müller, Paulo Nuin, Mark Quinton-Tulloch, Daniela Raciti, Tim Schedl, Gary Schindelman, Lincoln Stein

**Affiliations:** Division of Biology and Biological Engineering 140-18, California Institute of Technology, Pasadena, CA 91125, USA; Division of Biology and Biological Engineering 140-18, California Institute of Technology, Pasadena, CA 91125, USA; Division of Biology and Biological Engineering 140-18, California Institute of Technology, Pasadena, CA 91125, USA; Informatics and Bio-computing Platform, Ontario Institute for Cancer Research, Toronto, ON M5G0A3, Canada; Division of Biology and Biological Engineering 140-18, California Institute of Technology, Pasadena, CA 91125, USA; European Molecular Biology Laboratory, European Bioinformatics Institute, Wellcome Trust Genome Campus, Cambridge CB10 1SD, UK; Division of Biology and Biological Engineering 140-18, California Institute of Technology, Pasadena, CA 91125, USA; European Molecular Biology Laboratory, European Bioinformatics Institute, Wellcome Trust Genome Campus, Cambridge CB10 1SD, UK; School of Infection and Immunity, University of Glasgow, Glasgow G12 8TA, UK; Informatics and Bio-computing Platform, Ontario Institute for Cancer Research, Toronto, ON M5G0A3, Canada; Division of Biology and Biological Engineering 140-18, California Institute of Technology, Pasadena, CA 91125, USA; Division of Biology and Biological Engineering 140-18, California Institute of Technology, Pasadena, CA 91125, USA; Division of Biology and Biological Engineering 140-18, California Institute of Technology, Pasadena, CA 91125, USA; European Molecular Biology Laboratory, European Bioinformatics Institute, Wellcome Trust Genome Campus, Cambridge CB10 1SD, UK; European Molecular Biology Laboratory, European Bioinformatics Institute, Wellcome Trust Genome Campus, Cambridge CB10 1SD, UK; European Molecular Biology Laboratory, European Bioinformatics Institute, Wellcome Trust Genome Campus, Cambridge CB10 1SD, UK; School of Infection and Immunity, University of Glasgow, Glasgow G12 8TA, UK; Division of Biology and Biological Engineering 140-18, California Institute of Technology, Pasadena, CA 91125, USA; Informatics and Bio-computing Platform, Ontario Institute for Cancer Research, Toronto, ON M5G0A3, Canada; European Molecular Biology Laboratory, European Bioinformatics Institute, Wellcome Trust Genome Campus, Cambridge CB10 1SD, UK; Division of Biology and Biological Engineering 140-18, California Institute of Technology, Pasadena, CA 91125, USA; Division of Biology and Biological Engineering 140-18, California Institute of Technology, Pasadena, CA 91125, USA; Informatics and Bio-computing Platform, Ontario Institute for Cancer Research, Toronto, ON M5G0A3, Canada; European Molecular Biology Laboratory, European Bioinformatics Institute, Wellcome Trust Genome Campus, Cambridge CB10 1SD, UK; Division of Biology and Biological Engineering 140-18, California Institute of Technology, Pasadena, CA 91125, USA; Informatics and Bio-computing Platform, Ontario Institute for Cancer Research, Toronto, ON M5G0A3, Canada; European Molecular Biology Laboratory, European Bioinformatics Institute, Wellcome Trust Genome Campus, Cambridge CB10 1SD, UK; Division of Biology and Biological Engineering 140-18, California Institute of Technology, Pasadena, CA 91125, USA; Department of Genetics, Washington University School of Medicine, St. Louis, MO 63110, USA; Division of Biology and Biological Engineering 140-18, California Institute of Technology, Pasadena, CA 91125, USA; Informatics and Bio-computing Platform, Ontario Institute for Cancer Research, Toronto, ON M5G0A3, Canada

**Keywords:** genome annotation, knowledgebase, software, disease models, bioinformatics tools, model organism, Global Core Biodata Resources, *Caenorhabditis elegans*

## Abstract

WormBase has been the major repository and knowledgebase of information about the genome and genetics of *Caenorhabditis elegans* and other nematodes of experimental interest for over 2 decades. We have 3 goals: to keep current with the fast-paced *C. elegans* research, to provide better integration with other resources, and to be sustainable. Here, we discuss the current state of WormBase as well as progress and plans for moving core WormBase infrastructure to the Alliance of Genome Resources (the Alliance). As an Alliance member, WormBase will continue to interact with the *C. elegans* community, develop new features as needed, and curate key information from the literature and large-scale projects.

## Introduction

The early organization of knowledge about *Caenorhabditis elegans*, in the form of the *Worm Breeder's Gazette*, the Cold Spring Harbor Laboratory Press book *C. elegans* (Wood 1988), Worm Community System (Shoman *et al.* 1995), and Leon Avery's World Wide Web Server, coalesced around the genome into ACeDB (A *C. elegans* Data Base; Durbin and Thierry-Mieg 1994). WormBase was rebranded from ACeDB in 2000 when Lincoln Stein and Paul Sternberg joined Richard Durbin, John Spieth, and Jean Thierry-Mieg as leads of the project (Stein *et al*. 2001). Since then, WormBase has expanded in breadth and depth (Harris *et al*. 2003; Harris, Clark *et al*. 2004; Harris, Chen *et al*. 2004; Harris *et al.* 2010, 2014, 2020; Chen *et al.* 2005; Schwarz *et al.* 2006; Bieri *et al.* 2007; Rogers *et al*. 2007; Howe *et al.* 2012, 2016; Yook *et al.* 2012; Lee *et al.* 2018; Davis *et al.* 2022) to become an essential resource for researchers in *C. elegans*, many other nematodes, and the broader biological and bioinformatic communities (Baxevanis 2011; Tang *et al*. 2015). Over time, Thierry-Mieg and Spieth left the project; Durbin was replaced by Paul Kersey, Kevin Howe, and recently, Sarah Dyer. Professional staff has remained remarkably constant except at European Molecular Biology Laboratory (EMBL)-European Bioinformatics Institute (EBI), where the institute's underlying mission is to promote molecular biology research in Europe, train scientists, and ensure that their acquired knowledge and skills are returned to the scientific community. The Global Biodata Coalition (Anderson *et al*. 2017) has designated WormBase as a Core Global Biodata Resource, recognizing that WormBase is “…of fundamental importance to the wider biological and life sciences community and the long-term preservation of biological data.”

Over 20 years ago, several model organism databases (MODs) formed the Gene Ontology Consortium (GOC) to derive a common language to describe gene function, and WormBase joined the consortium shortly thereafter in 2003 (Harris, Clark *et al*. 2004; Harris, Chen *et al*. 2004). This common ground set the stage for the formation of the Alliance of Genome Resources (the Alliance) in 2016 when several MODs joined forces to plan and implement shared infrastructure (Alliance of Genome Resources Consortium 2022 ; Alliance of Genome Resources Consortium 2024). There are 3 compelling reasons for WormBase to transition its infrastructure to the Alliance (Alliance Central). First, while WormBase is an exemplary member of the MOD community, there is considerable room for improvement. Second, WormBase funding has been reduced to <50% of 2010 levels not counting inflation, and thus, the current level of service is not sustainable on its own. Third, the MODs currently do not provide standardized, facile services to those interested in comparing genes across major biomedical models (e.g. worm–human–fly–yeast–fish) an increasingly important, if not essential, feature as comparative information expands. Given this critical juncture in the history of WormBase, we choose to focus on our transition to the Alliance in detail here, as well as to discuss the current state of WormBase for context.

## Infrastructure moving to the Alliance

For every MOD, there is a need to balance rapid data updates with stable, reliable infrastructure. WormBase started with a database (ACeDB) using the acedb data management system, which was built for handling the *C. elegans* genome project, the first animal genome to be completed (*C. elegans* Sequencing Consortium 1998; Hillier *et al.* 2005). In addition, acedb allowed seamless integration of complex data stored in different instances, for example, 2 separate genome databases corresponding to the then Sanger Center and Washington University Genome Sequencing Center. During 2017–2020, we transitioned our data storage to Datomic, another nonrelational data management system (Davis *et al.* 2022). The flexibility of our system and processes allowed flexible and rapid data modeling but eventually needed cached data to allow the website to perform fast enough and a lengthy build process that slowed the process of propagating corrections to the website. On the positive side, we built the website in a highly modular fashion with individual widgets that could obtain data from ACeDB, Datomic, or other data sources such as flat files on servers. This architecture allowed the migration from acedb to Datomic to be opaque to users, and we expect it will help smooth the less opaque migration to the Alliance.

The Alliance started with a graph database system (Neo4J) that allowed data loading from individual MODs and running the new website; it was a flexible solution to rapidly get a common portal in place. This then gave time to plan a long-term solution for harmonized data at the Alliance, in the form of a PostgreSQL relational database. This architecture means new information can be propagated to the website rapidly, although whether this is continual or in monthly releases will depend on the type of information. This more rapid propagation of information from curated knowledgebase to website will be better for users. One potential downside to shared MOD infrastructure is decreased responsiveness to new features due to more requirements for harmonization, but the modular design of the Alliance system and the increased number of biocurators and software engineers in the now larger overall project will facilitate timely deployment of improvements.

The data currently loaded into the Alliance Neo4J database represent the initially harmonized data types from WormBase: a subset of all data types and, within a data type, a subset of data type properties. These data include genes (including basic gene information like name, symbol, synonyms, and descriptions), alleles and variants (including transgenic alleles and their constructs), models (including strains and genotypes), gene expression annotations (low- and high-throughput), disease model annotations, phenotype annotations, and interactions (molecular and genetic). Data type harmonization for the remaining data types and properties is ongoing among Alliance members, and the resulting harmonized data are being deposited into the persistent PostgreSQL database to act as the primary curation database and eventual source of truth for Alliance-curated data. Over the next several months, Alliance software developers will be transitioning the source of data on the public Alliance website from Neo4J to the persistent PostgreSQL store and will begin providing newly harmonized representations of data not already present on the site. WormBase staff will continue to provide the aforementioned data to the Neo4J database (until its retirement) and in LinkML format (see below) to the PostgreSQL persistent store, including all more recently harmonized data types and properties. Additional fully harmonized data types to be imported into the PostgreSQL database and, eventually, the Alliance website include reagents (e.g. clones and antibodies), genome features (e.g. DNA binding sites, enhancers), RNAi phenotype annotations, regulatory interactions (e.g. geneA upregulates expression of geneB), transcripts and proteins, chromosomes and genome assemblies, transcription factors, expression clusters (sets of coregulated genes), gene site-of-action data, and person and laboratory data (representing the research community). Over the next year, the Neo4J-to-PostgreSQL database migration should be completed and the Alliance should begin offering newly harmonized data in the Alliance public website sourced from the persistent curation database. Completion of all remaining WormBase data harmonization, import, and representation on the Alliance website will likely span the next 2 years.

At this juncture, it is useful to discuss each major data type in WormBase and its status with respect to inclusion in the Alliance. As the Alliance platform continues to be developed, additional WormBase data types will systematically be integrated. Currently, a subset of data is regularly imported from WormBase to the Alliance. Moving forward, the WormBase site will continue to be supported until the transition to the Alliance is complete.

## Genome

The genome is first and foremost. Of the Alliance members, only WormBase, SGD, and FlyBase are responsible for the ownership of their genomes; however, maintaining the genome is a priority for WormBase and remains crucial for emerging genome communities as well as other MODs that may join the Alliance in the future. We will switch genome sequence and feature curation from ACeDB to Apollo (Dunn *et al.* 2019), which will be more accessible to researchers wishing to improve sequence feature annotations on their favorite nematode (e.g. Moya *et al.* 2023).

### Genes structure models

The set of 19,984 protein-coding genes and 28,032 ncRNA genes (in release WS291) are fundamental to most analyses with *C. elegans*. There is only a slow change in gene number and precise structure of introns and UTRs (see Yoshimura *et al*. 2019). Gene structure curation will continue to be handled by WormBase per se.

### Genetic loci

Of the 19,984 protein-coding genes in *C. elegans*, 10,838 have a name/gene symbol (e.g. *lin-12*). Gene naming is largely investigator-initiated, with a request to WormBase through genenames@wormbase.org ; this will continue with *C. elegans* content in the Alliance. The gene symbol, which is formatted in lowercase and italics, communicates information about the gene based on mutant phenotype, functional criteria, orthology, or homology. To make genes easily recognizable to non-*elegans* researchers, the current preference is to name genes with human orthologs after the human gene if characterized. Gene name stability and formatting are important to avoid confusion in the literature and to facilitate searches of other databases that use the *C. elegans* gene symbols (e.g. UniProt) and text mining. However, occasionally names have been changed because of incorrect orthology or not supported by functional studies from the community. Names have also been changed based on requests from multiple community members (e.g. *daf-21* renamed as *hsp-90*). To avoid name changes and to have the published name used in databases, researchers should contact WormBase prior to manuscript submission; the most common issue is that the requested name is already in use in *C. elegans* or another model organism for a nonhomologous gene product. Over the past 10 years, the community has averaged 180 new gene names per year, often with a corresponding publication, increasing knowledge about our favorite organism. More information about *C. elegans*–specific nomenclature can be found at https://wormbase.org/about/userguide/nomenclature.

### Orthologs and paralogs

WormBase.org displays orthology from the Ensembl Compara pipeline. https://useast.ensembl.org/info/about/publications.html. The Alliance has orthology inferences from the Quest for Orthologs (Nevers *et al.* 2022; discussed in Alliance of Genome Resources Consortium 2024); the Alliance ortholog set allows comparison among the model organisms in a reciprocal manner. If there are any specific issues with ortholog assignments, the Alliance can manually modify assertions as they do for ZFIN with curator-chosen orthology assertions. The Alliance recently added paralogs, a useful feature of the WormBase homology section. A next step will be to add comparisons of expression, disease models, and function among the paralogs using the Ribbon approach (a feature currently available for orthologs); this will be a new feature for WormBase users. With these changes, switching to Alliance orthology inferences will be beneficial to users.

### Genome features

**Fig. 1.**
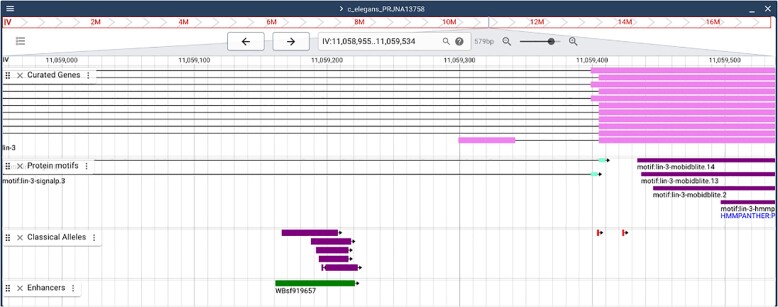
JBrowse with sequence features. A partial view of the *lin*-3 sequence in the JBrowse viewer. “Classical alleles” (amusingly including CRISPR gene edits) shown in purple provide evidence for the *lin-3* vulval enhancer (*WBsf919657*) shown in green. The top shows the displayed coordinates and allows navigation with left and right arrows as well as the ability to zoom in or out. Users can choose the tracks that are displayed, with many choices hidden in the menu at the upper left. Here, curated exons are shown (magenta) (*lin-3* has alternative 5′ exons, one of which is shown here) in the intron of majority transcription unit. Protein motifs are shown next (purple), followed by mutant alleles and finally an enhancer (green).

In addition to gene structure models, features such as binding sites and enhancers are annotated in Gene Feature Format (GFF3) files and displayed on JBrowse 2 (Diesh *et al*. 2023) and on genome feature pages (Fig. 1). Discussions on harmonizing descriptions for genome features at the Alliance are ongoing, but the existence of standard formats and the Sequence Ontology (e.g. Sant *et al.* 2021) suggest this approach will work well.

### Variants and alleles

Alleles with phenotypes, alleles with no associated phenotypes, natural variants, and other polymorphisms are curated at WormBase and can be located under the “Genetics” widget of a gene page. In the Alliance, WormBase “alleles” are classified into 3 variant classes: “phenotypic variants,” ­where an allele has a genomic location overlapping a single gene and has been associated with a phenotype; “alleles,” where there is no associated phenotype and the genomic location may or may not be known; or “high-throughput variants,” which have genomic locations but no associated phenotypes and include natural variants. All WormBase alleles that are considered “phenotypic variants” have been migrated to the Alliance.

Both WormBase and the Alliance run the Ensembl Variant Effect Predictor tool (McLaren *et al.* 2016) to generate predicted variant consequences and Human Genome Variation Society (HGVS) nomenclature (den Dunnen 2017), with missense variants being further annotated with predicted pathogenicity scores using PolyPhen-2 (Adzhubei *et al.* 2013) and SIFT (Kumar *et al.* 2009).

### Transgenes and constructs

Transgenes and constructs include integrated and extrachromosomal objects created by the worm community to study, for example, gene function and expression. The construct class records the individual engineered genomic snippets that are used to create the transgene in the worm. In the Alliance data modles, transgenes are considered to be a type of variant because transgenes are treated as engineered alleles by other members of the Alliance. This change in classification should not have any impact when looking up these objects in WormBase or in the Alliance; however, when searching the Alliance for these objects in batch, users may need to start their search from the variant class. For now, transgenes and constructs will continue to be annotated as needed for the curation of other data types, such as phenotype and gene expression.

### Strains

Strain information in WormBase is added and updated, from periodic data transfers from the Caenorhabditis Genetics Center (CGC) (Tuli *et al*. 2018) as well as by curators based on published papers. In addition, the adoption of disease model curation, where strains are curated as models of disease, helped to formalize strain curation in WormBase, e.g. strains are assigned a WBStrain ID for unambiguous identification like other data objects such as transgenes and variations. Strains are curated as part of phenotype, gene expression, and disease curation.

### RNAi

WormBase has routinely remapped RNAi reagents for each release. Going forward, this will be done annually. Phenotypes associated with RNAi experiments are not yet in the Alliance but will be added in about a year, after the treatment of sequence-based reagents (e.g. RNAI, morpholinos) is harmonized.

### Antibodies

WormBase maintains a curated list of antibodies produced from individual laboratories. We collect antibody-related information from the literature and from direct researcher submissions. This information includes details such as the antibody name, target antigen, species specificity, experimental methods, and associated references. The Alliance data model for the antibody class is harmonized, and the entire collection of WormBase antibodies will be incorporated into the Alliance within a year.

### Chemicals and small molecules

This data class includes drugs, chemicals, naturally occurring biological compounds, and metabolites. These entities are captured during disease, phenotype, large-scale gene expression, and interaction curation. Where possible, the published name used by the author is matched by name or the Chemical Abstracts Service Registry Number (CasRN; http://www.cas.org) with an entry in the Chemical Entities of Biological Interest ontology (ChEBI; http://www.ebi.ac.uk/chebi/; Degtyarenko *et al*. 2007). However, it is not uncommon for the chemical to lack a ChEBI ID in which case it is matched with an ID from the Comparative Toxicogenomic Database (CTD; http://ctdbase.org/; Davis *et al.* 2021) using name, medical subject heading (MeSH) vocabulary IDs, or CAS RN. Synonyms of the chemical name are retrieved from both ChEBI and the CTD for easier retrieval by curators and WB users. We will continue to capture chemicals and small molecules as part of curation workflows. These entities are due to be imported into the Alliance and harmonized with chemical and small molecule data sets curated by other Alliance members during the next year.

### Phenotypes

Phenotype data comprise all annotations that include terms from the *C. elegans* Phenotype Ontology (https://github.com/obophenotype/c-elegans-phenotype-ontology). Phenotypes are annotated to genes based on results reported from perturbations (RNAi knockdown, mutant analysis, or gene overexpression) as well as gene interaction experiments. Phenotypes are also annotated to rearrangements and strains that might have multiple genetic differences from the reference strain; when these data are migrated to the Alliance, phenotype annotations will be included. Phenotypes of genes studied as models of human disease are already included in the Alliance.

### Gene expression

WormBase expression data encompasses single-cell and bulk RNA-seq data derived from tissues or entire organisms, in addition to manually curated experiments examining individual gene expression profiles and cell type–specific expression patterns. Data are accessed via the “Expression” widget on the gene page. The widget's tables and displays bring together comprehensive evidence regarding the expression of the gene within a specific anatomical entity, life stage, or cellular component. This widget offers a quick overview of the number of independent experiments that corroborate the gene’s expression at various times and locations.

Low-throughput expression data displayed on the WormBase widget is also presented on the Alliance gene report pages, with few notable differences. The initial set of metadata that is included on the Alliance website includes a minimal set of information to represent the spatiotemporal location of the gene product, such as the gene under study, the anatomical structure, the life stage, and the cellular component where the gene was found to be expressed. Additional information about experimental details, such as the reagents used (antibodies, knock-in alleles, and reporter gene constructs) and audiovisual material (movies and images), is currently available via a link to WormBase and will be integrated into the Alliance website incrementally in the near future. Importantly, the harmonization process that is necessary to make the related information in 6 different organisms compatible is almost completed for these ancillary metadata. It is important to highlight that accessing expression data through the Alliance website provides the valuable capability of obtaining a unified perspective on gene expression for human, mouse, rat, frogs, zebrafish, fruit fly, and yeast. This can be achieved by selecting the “Compare ortholog genes” checkbox.

For high-throughput expression studies, users can browse and download data from SPELL (https://spell.wormbase.org/), the Expression Dataset Locator (https://wormbase.org/tools/rnaseq/expression_dataset_locator.cgi), and the RNA-seq FPKM Search (https://wormbase.org/tools/rnaseq/fpkmmine.cgi) tools (Harris *et al.* 2020). We annotate differentially expressed genes of each study as expression clusters. The expression clusters include information about tissue enrichment, drug or treatment response, and gene regulation on each gene page. Tissue enrichment data of expression clusters, including those derived from single-cell RNA-seq studies, are also displayed in the WormBase Ontology Browser. These displays are based on data that will take longer to harmonize and will thus be available on the “Expression” section of the Alliance in about 2 years, after other expression data have been migrated.

We also expanded the set of single-cell RNA-seq data sets displayed on the “Expression” widget of gene report pages, adding to the one generated by the CeNGEN consortium ([Bibr iyae050-B52][Bibr iyae050-B52]). We now display a single-cell transcriptional profile from *C. elegans* embryos (Packer *et al*. 2019; Kishore *et al*. 2020) and are planning to add additional studies. These single-cell graphs will also be displayed on the Alliance gene expression detail pages currently under development.

### Interactions

The ability to study interactions between genes and gene products is a great strength of *C. elegans* as an experimental system because of the ease of combining mutations due the male-hermaphrodite mode of reproduction, the existence of 6 linkage groups, and enough progeny to obtain recombinants. Biocurators at WormBase extract assertions of gene–gene interaction from the literature and supplemental tables of papers. These are currently sent to the Alliance for display. The concepts of gene interaction (e.g. Huang and Sternberg 2006) have mostly been harmonized and will be added to Alliance data store in the future. An Alliance interaction viewer, such as the Venn diagram tool, Vennter (Cho *et al.* 2020) will be implemented within 2 years.

### Gene Ontology annotations

The Gene Ontology (GO) is designed to be a multispecies ontology (Gene Ontology Consortium 2023), and thus, GO annotations were naturally included in the Alliance from the beginning. GO annotations displayed at the Alliance are retrieved from the GOC infrastructure; up-to-date ontology information is retrieved similarly. GO annotations at the Alliance are initially displayed in GO ribbons that summarize annotations using an Alliance subset (“slim”) and allow a comparative view. GO annotations may also be viewed in a table format that includes supporting evidence. The main limitation of GO ribbons is that the high level subset used is not tuned to nematodes. Eventually we will create nematode-centric choices to complement the generic GO slim. In addition to a GO ribbon and table display, WormBase also has a tool for visualization annotations to GO terms that simplifies the ontology on a case-by-case basis, for example, displaying enriched terms (SObA graphs, Lee *et al.* 2018). A general Alliance enrichment tool is in the planning stages; inclusion of the SObA graphs to visualize enriched terms will come at a later date.

### People and labs

WormBase has basic information about nematode researchers (people) that not only facilitates communication with users but also allows us to connect individuals to gene classes, references, allele classes, and their intellectual lineage. In addition, the “community curation” contributed by each person is acknowledged on their person pages in WormBase. Person curation with its associated information will be added to the Alliance incrementally over the next year. For the *C. elegans* communities, Laboratories are connected to people, strains, gene classes, alleles, and laboratory websites. One of the current priorities for the Alliance is to produce pages describing each paper and the entities to which they are connected. This latter work should be completed by the end of 2024.

### Genetic pathways

WormBase has previously not devoted substantial attention to curating genetic pathways, in part because a suitable framework had not been developed. The Gene Ontology Causal Activity Models (GO-CAM) models now provide an appropriate framework (Thomas *et al.* 2019). In these models, the annotation for individual gene products is linked so that a protein has a molecular function in a particular cellular component with specific inputs and outputs with defined relations in the context of a specific biological process; this information allows pathways to be constructed and displayed in a consistent manner.

**Fig. 2.**
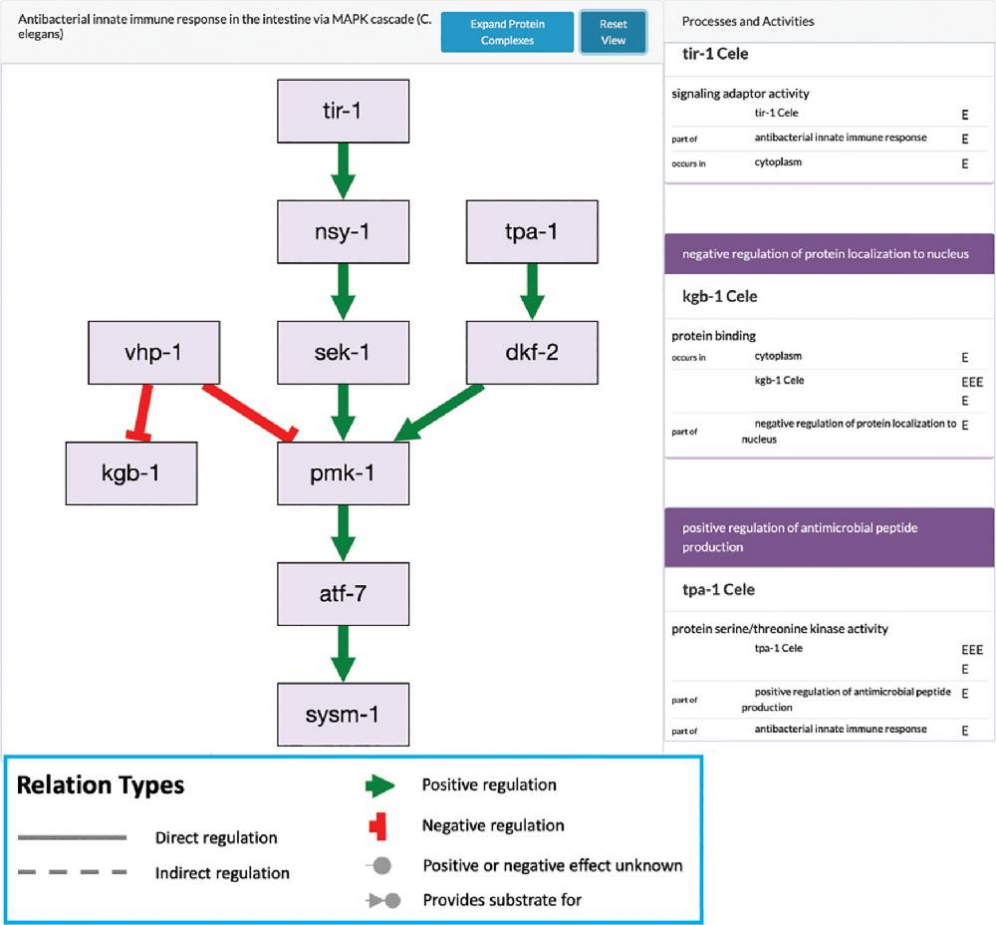
Example of a *C. elegans* genetic pathway at the Alliance. This pathway diagram for PMK-1 signaling is generated automatically from a curated GO-CAM pathway. In this example, only direct positive and negative regulation occur, but the underlying model can handle indirect inputs as well as biochemical pathways using chemicals as reaction inputs and outputs. The rectangles on the right side show the standard GO annotations associated with the GO-CAM, including molecular function, cell component, and biological processes.

While the number of curated GO-CAM pathways is still relatively small, it is growing, and pathway views can be generated from the underlying data curated by WormBase and displayed at the Alliance using software (Fig. 2). The “Pathways” widget on the Alliance gene pages also shows projected reactome pathways based on curated human models (Milacic *et al.* 2024). The curated GO-CAM pathways are expected to improve gene set enrichment analysis (e.g. Markarian *et al.* 2024).

## WormBase UI

### Homepage and static pages

**Fig. 3.**
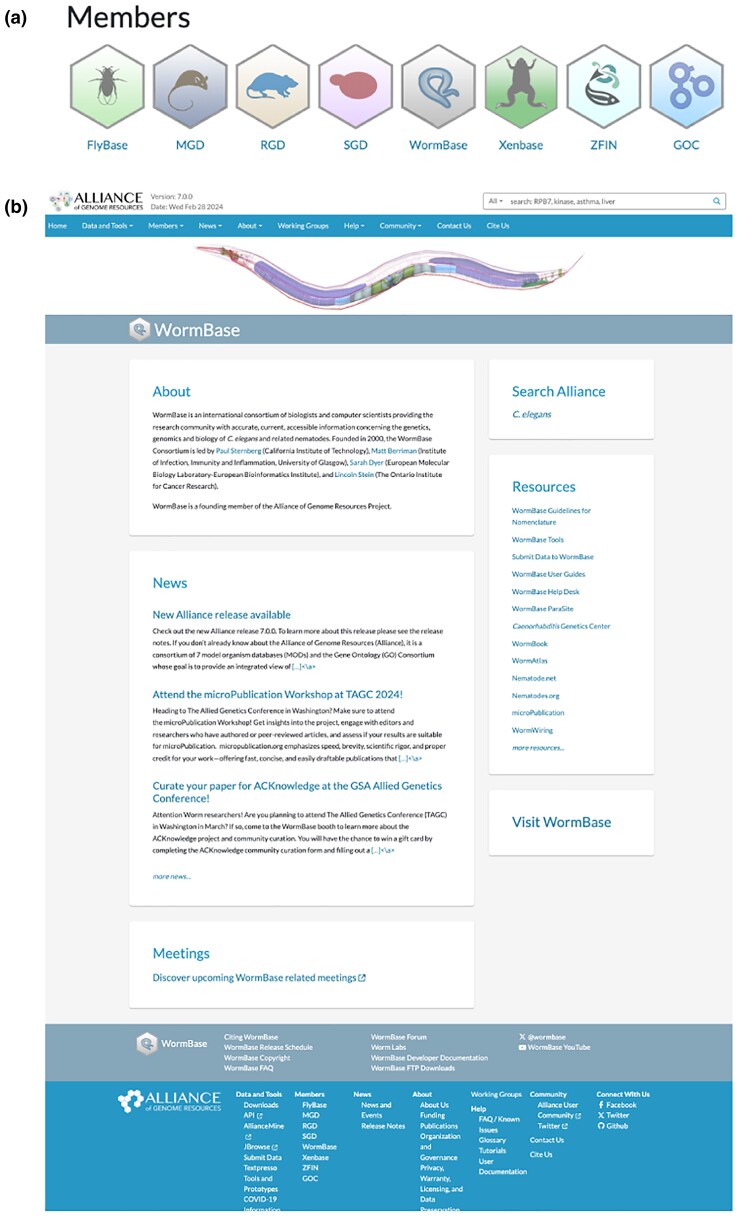
A home for *C. elegans* at the Alliance. a) MOD icons at the bottom of the Alliance homepage. b) WormBase homepage at the Alliance.

Many users start their interaction with WormBase.org via the homepage to search or open tools such as WormMine or multi-ontology enrichment analysis. The Alliance has a generic homepage, from which one can click on the WormBase hexagon (Fig. 3a) to go to the WormBase homepage at the Alliance (Fig. 3b) and, if desired, to the current WormBase.org (“Visit WormBase”). In addition, from any gene page, one can click on the equivalent WormBase gene page. The many static pages that have useful but relatively unchanging information (such as a guide to Nomenclature) will be linked from the WormBase.org homepage. These links will be added to the Alliance WormBase page in the coming year.

### Gene page

**Fig. 4.**
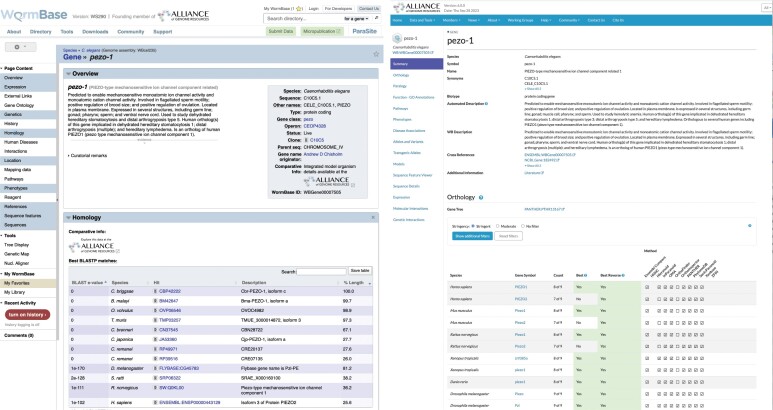
Comparison of WormBase and Alliance gene pages. Shown is *pezo-1* with the WormBase view on the left and Alliance view on the right.

Gene pages are complex, with access to a host of information. In general, the information will be present in both WormBase and Alliance views but organized a bit differently (Fig. 4). WormBase gene pages allow customized user ordering of different widgets, and this is not yet available at the Alliance. On the other hand, at the Alliance, the information on different organisms is organized similarly to facilitate cross-organism comparisons.

## WormBase tools

A summary of the major WormBase tools and displays as well as their migration status is given in Table 1.

**Table 1.** Tools in WormBase and status. For each tool, the current location if not WormBase.org is listed as is the destination and estimated time of migration.

Tool	Current location(s)	Migration	Eventual destination
FTP site	downloads.wormbase.org, Hinxton	1 yr	Alliance
GBrowse		No	Alliance
JBrowse		Done	Alliance
Forums	forums.wormbase.org	Done	Alliance-hosted WB portal
Wiki	wiki.wormbase.org	2 yr	Alliance-hosted WB portal
Blog	blog.wormbase.org	1 yr	Alliance-hosted WB portal
AQL/WQL gateways		Never	Alliance-hosted WB portal
API		2 yr	Replaced by Alliance
SPELL		1 yr	Alliance-hosted WB portal
SimpleMine		Partly done	Alliance
WormMine	intermine.wormbase.org	1 yr	Alliance
BLAST/BLAT		Done	Alliance
ePCR		2 yr	Alliance
Name Service		1 yr	Alliance
Apollo		2 yr	Alliance
Mailing lists		1 yr	Alliance
Email accounts		1 yr	Alliance
Nucleotide Aligner		Replaced 1 yr	Alliance
Protein Aligner		Replace 1 yr	Alliance
Ontology Graphs		2 yr	Alliance or Caltech server
Ontology Enrichment		1 yr	Alliance

### JBrowse

Over the last year, JBrowse, one of the most widely used tools provided by WormBase, has undergone an overhaul into JBrowse 2 (Diesh *et al*. 2023) and has been implemented at WormBase and moved to Alliance infrastructure. This new version of JBrowse provides the functionality of JBrowse 1 with the addition of several new features. In addition to the linear genome view (i.e. the traditional genome browser view of a region of a single stretch of genome with data tracks), JBrowse 2 provides other data views, including linear genome views that span multiple chromosomes (up to an entire genome), whole genome comparisons in the form of dotplots, pairwise synteny views, a “spreadsheet” view, and views that allow exploration of structural variants. JBrowse 2 also provides the ability to create scalable vector graphics output of all views, which was by far the most requested JBrowse feature.

**Fig. 5.**
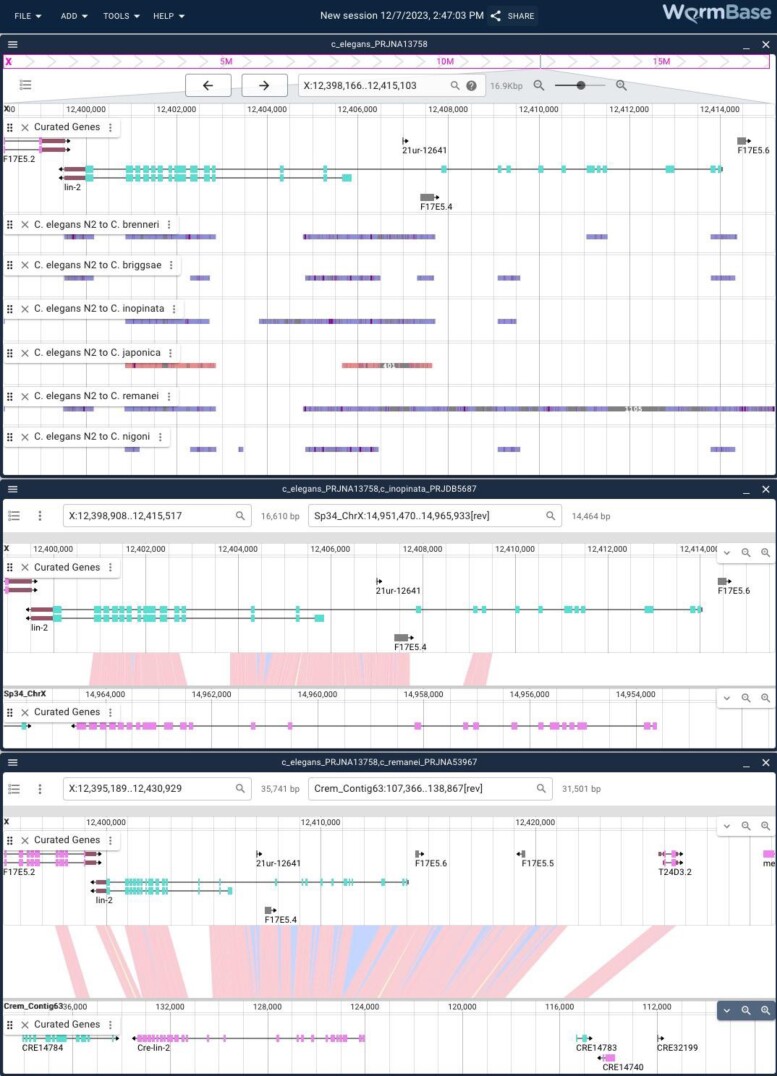
Linear genome and synteny views in JBrowse 2. JBrowse 2 showing multiple views: the top panel is a linear genome view showing the region around the *C. elegans  lin-2* gene with multiple synteny tracks showing the sequence similarity as determined by minimap2 analysis between the N2 assembly and the assemblies of multiple related genomes. The middle panel is the result of right clicking one of the regions showing synteny between *C. elegans* and *Caenorhabditis inopinata* and zooming out slightly to show the entire *lin-2* in both assemblies. The lower panel is the same but with the similar region in *C. remanei*.

JBrowse 2 supports comparative genomics data from a variety of established comparison tools like minimap2 (Li 2018) and MCScanX (Wang *et al.* 2012). WormBase has provided several precomputed pairwise genome comparisons, primarily between several of the Caenorhabditis assemblies, using minimap2 to perform the analysis (Fig. 5). More comparison data sets can be added on request from users, and as with all supported views in JBrowse 2, “session” tracks that are accessible just for the user can be created with their own data and analysis results. These data are available both as views created from the “New Session/Launch View” interface as well as from a linear genome view by adding any of the tracks from the “Synteny” category of tracks.

Added functionality with JBrowse 2 includes several tools for personalizing a user's interaction with it. In addition to the ability to add session tracks, users have the ability to use the spreadsheet view to load a list of regions of interest that can be quickly navigated to in succession. Similar functionality is the ability to create and edit bookmarks of regions that have been visited and the user wants to save. These bookmarks can also be exported as a tab separated file that can be uploaded later and shared with other users. Since JBrowse 2 can create very complicated displays with multiple views, it also provides a way to create URL-shortened share links that will save the exact view layout that can be easily shared with collaborators. Finally, there are several help documents for WormBase users new to JBrowse 2 that are available under the “Help” menu.

## Gene summaries

WormBase continues to generate and update gene summaries that are text descriptions of gene function. These summaries are generated for 10 nematode species based on structured, curated data in the WormBase database using an algorithm developed at WormBase and further refined for the Alliance (Kishore *et al*. 2020). Gene summaries cover 5 Caenorhabditis species (*C. elegans*, *Caenorhabditis brenneri*, *Caenorhabditis briggsae*, *Caenorhabditis japonica*, *Caenorhabditis remanei*) and 5 parasitic species (*Onchocerca volvulus*, *Pristionchus pacificus*, *Strongyloides ratti*, *Trichuris muris*) and can be viewed in the “Overview” section at the top of gene pages. For well-studied genes, manually curated data are the main source of information, while high-throughput data are largely used for less studied genes. Summaries for genes for the Caenorhabditis species such as *C. japonica* and *C. remanei* are generated by relating them via orthology to *C*. *elegans* genes. As part of the ongoing WormBase migration process to the Alliance, we will (1) integrate the Alliance and WormBase gene summary pipelines for *C. elegans* and (2) explore the use of AI for writing gene summaries for those genes that are less studied and/or have fewer structured, curated data annotations in WormBase. As part of this process, we will customize and finetune large language models (LLMs) to generate high-quality and scientifically rigorous gene summaries which will help scale gene summary generation to cover the fast-growing literature.

## *C. elegans* models of human disease

*C. elegans* is increasingly being used to model human disease. Modeling takes the form of expressing human proteins (or parts thereof) in *C. elegans*, studying phenotypes that are the phenologs (McGary *et al.* 2010; Golden 2017) of human disease phenotypes, studying orthologs of human disease-relevant genes, or studying disease-associated variants either in the orthologous protein (e.g. Wong *et al.* 2019) or in a humanized worm (e.g. McDiarmid *et al.* 2020). WormBase continues to curate such models of human disease from papers by associating genes, alleles, genotypes, and strains with a term from the Disease Ontology (DO) (Schriml *et al.* 2022) according to annotation standards defined by the Alliance. Other experimental data such as drugs, herbals, small molecules, and other compounds that act as inducers (induce disease-like phenotypes) and/or modifiers (ameliorate or exacerbate the disease condition) are also included in an annotation. Note that as part of the migration of WormBase data and displays to the Alliance, disease-related data displays are actively being developed and are almost complete at the Alliance. Users are encouraged to view and download these data on gene and disease pages at the Alliance where harmonized worm disease data can be viewed in the context of other species.

## WormBase literature acquisition and processing

The goal of the WormBase literature acquisition and processing pipeline is to identify in PubMed all references about *C. elegans* and include them in the WormBase bibliography. We perform a daily PubMed query using the keyword “elegans,” and a curator manually reviews the results to accept appropriate references for inclusion in the WormBase corpus. For all approved references, we attempt to retrieve the full text and associate entities, e.g. species, genes, alleles, transgenes, and 15 different data types, e.g. RNAi phenotypes, genetic interactions, and anatomical expression patterns, with the reference via pattern matching and machine learning algorithms, such as neural networks, respectively. Curators use the results of the machine learning algorithms to prioritize references for curation. In addition, we use pattern matching followed by manual validation to associate a reference's authors to a WormBase “person,” e.g. Iva S. Greenwald is connected to WBPerson220, to populate the publications section of each Person page in WormBase.

WormBase is systematically transitioning our literature acquisition and triage pipelines to Alliance literature system infrastructure, known as the Alliance Bibliography Central (ABC). The daily PubMed search, sort (i.e. acceptance into the WormBase literature corpus), species assignments, and full-text acquisition and storage are implemented in the ABC. Current work is focused on the final phase of testing the Alliance reference data model for data type and entity tagging, specifically with respect to sufficiently capturing the provenance of paper tags (i.e. manual assertions vs machine learning methods) and, wherever possible, automatically using manual assertions to validate machine learning methods.

For the Alliance reference tagging data model, we worked with editors of the Evidence and Conclusion Ontology (ECO) (Nadendla *et al.* 2022) to generate new evidence codes to capture the automated methods we use for paper tagging. In addition to adopting a unified literature acquisition and processing system, the transition to the ABC will afford WormBase, and other Alliance member MODs, a way to coordinate curation for multispecies papers, a currently ad hoc process, if done at all. A unified literature system will also allow WormBase to share its machine learning methods with other Alliance members as well as benefit from methods developed by other groups. The end result will be faster, more systematic literature curation across a broad group of model organisms, not just *C. elegans*.

### Ontologies

Ontologies are an essential aspect of accurate and consistent biocuration. WormBase uses community standard ontologies for curation, and we maintain several worm-specific ones, such as anatomy, life stage, and phenotype. We will continue to maintain the worm-specific ontologies to support curation and other usage demands, e.g. enrichment analysis (see below).

We use the WormBase Ontology Browser to display terms, their logical relationships, and annotation summaries for each ontology. This ontology browser is a stand-alone application built on the software stack of AmiGO, the GOC's ontology browser. The Alliance is planning a new ontology browser, which will replace the WormBase one.

### Gene set enrichment

WormBase hosts a suite of enrichment analysis tools based on the curated data of gene expression, gene function with Gene Ontology, and gene mutant phenotype analyses (Angeles-Albores *et al.* 2016, 2018). These tools use up-to-date data from each WormBase release using the same software stack that drives the Ontology Browser. Since enrichment analysis is applicable to all MODs, the Alliance will prioritize the development of a state-of-the-art tool which will include data from WormBase and the other MODs. In the interim, we will continue to support the existing tool at WormBase.

### SimpleMine

SimpleMine allows biologists to readily retrieve essential information for lists of genes. To facilitate ID mapping, SimpleMine has a built-in function to convert 9 types of gene names or IDs to and from WormBase IDs (including MODs, public names, NCBI, PANTHER, Ensembl, and UniProtKB identifiers). Users can choose to retrieve up to 34 types of data related to expression, phenotypes and interactions, chromosomal locations, human disease, and functional annotations. The results are presented as 1 line per gene. The output can be either HTML or a tab-delimited file, and there is an option to merge duplicate gene entries. A new feature for WormBase SimpleMine is the ability to batch download chromosomal coordinates and transcription factors within 2 kb of the promoter regions of each gene.

We also developed an instance of SimpleMine for the Alliance for researchers to batch download essential gene information for *C. elegans*, yeast, fly, Xenopus, zebrafish, mouse, rat, and human. This Alliance SimpleMine (https://www.alliancegenome.org/agr_simplemine.cgi) provides easy gene name/ID conversion. Users can retrieve summarized anatomic and temporal expression patterns, variants, and genetic and physical interactions. Other essential gene information includes disease association and orthologs among all 9 species. As additional types of data are transferred to the Alliance, SimpleMine will be able to retrieve them.

### Ontology term pages

**Fig. 6.**
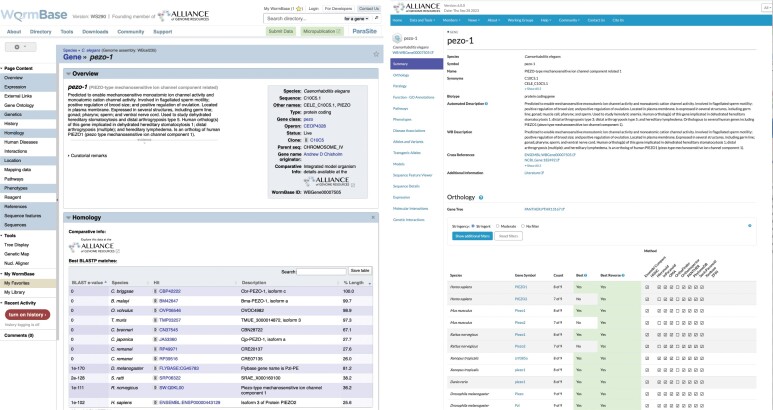
Mockup of an Anatomy Term page for “intestine”.

Ontology-based annotations are created by associating a genetic entity (e.g. gene, allele, genotype, strain, and transgene) with 1 or more terms in an ontology. We previously implemented ontology term pages at the Alliance for terms from the DO that include term definitions, synonyms, cross references to external resources, associated genes (human and all model organisms in the Alliance), associated alleles, and associated experimental models. We will soon extend this paradigm to other ontologies used at the Alliance including species-specific phenotype and anatomy ontologies. For example, anatomy ontology term pages will include sections for associated information including definitions, links to anatomy atlases, associations with genes and gene expression (including single-cell RNA-seq), disease associations, and images (Fig. 6). These will replace the anatomy term pages at WormBase.

## Community curation

WormBase remains committed to actively involving authors in data contribution via the collaborative process of community curation. We employ several approaches to facilitate author contributions. First, we have data type–specific, structured online submission forms that allow authors to contribute detailed experimental data. These forms notably include phenotype and allele sequence submissions. Second, we have a pipeline for authors to identify data in their newly published papers. The pipeline, initially known as Author First Pass (AFP; Harris *et al.* 2010) and recently enhanced into ACKnowledge (Author Curation to Knowledgebases; Arnaboldi *et al*. 2021), employs natural language processing and machine learning to identify biological entities and data types (for example, genes and anatomic expression patterns, respectively) from recently published research articles. Data extracted from the paper are presented to authors via a dedicated web interface where they can validate the results and, if desired, provide additional experimental details, including filling out the data type–specific forms mentioned above. Precision of entity recognition is enhanced by employing strategies such as tf-idf (term frequency–inverse document frequency, using the WormBase bibliography) or threshold values to predict entities experimentally studied in the paper and exclude entities, e.g. commonly used genetic markers, that are not the focus of the research. These strategies help to restrict the references section of web pages to the most relevant papers for a given entity. To allow authors to readily access all of their publications processed by the ACKnowledge pipeline and thus contribute curation at their convenience, we also recently implemented an Author Curation Portal (https://acp.acknowledge.textpressolab.com) where authors can enter their email address for access to curation forms for all of their published papers that have been processed by the ACKnowledge pipeline.

**Fig. 7.**
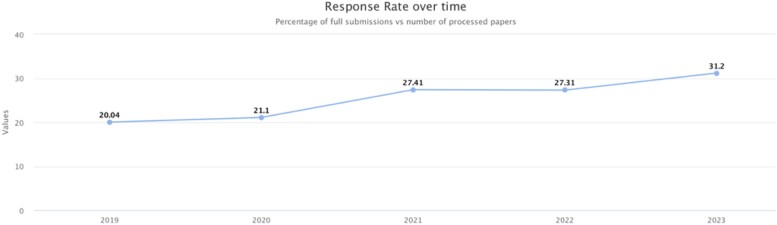
Community curation. The authors' response rate increased from 20% in 2019 to 31% in 2023, indicating that our efforts in community engagement are increasing participation.

The author response rate for the period September 2022 to September 2023 has risen to ∼31%, marking a notable increase from the previous 27% and continuing the trend of steadily increasing author participation (Fig. 7).

This improvement hopefully reflects our ongoing efforts to streamline the curation process, proactively engage authors, and provide incentives for their participation. For example, we launched a public contest for the June 2023 International Worm Meeting in Glasgow offering prizes for top community curation contributors, both for ACKnowledge and phenotype curation. The initiative was very successful and brought together researchers from around the world to contribute their expertise and knowledge to WormBase, promoting a sense of collegiality amongst our user community. We plan to continue awarding prizes to top community curators to recognize them for their valuable contributions. We also recognize community contributions in the “Activity” widget of the WormBase homepage by featuring the top 10 community curators, and we publicly acknowledge author contributions on multiple WormBase pages including the WormBase Paper and Person pages. Lastly, we send a personal thank you email once authors complete and submit the forms.

As WormBase transitions its literature infrastructure to the Alliance, we are actively engaged with the Alliance literature working group to transfer ACKnowledge processing and curation pipelines to the Alliance to effectively combine the capabilities and resources of both projects for mutual benefit and increasing author participation in the curatorial process.

## Conclusion

We look forward to more extensive discussions with users about how best to manage the major, but positive, transition of WormBase to the Alliance. Please be comforted that the existing WormBase website will remain operational well after the full transition to the Alliance, much as we did a decade ago when we moved to the current website infrastructure. Nonetheless, we are excited to offer the *C. elegans* community an enriched and exciting new experience at the Alliance where *C. elegans* research will be presented in the wider context of multiple model organism, and human, biomedical research. The present version of the Alliance site is focused on users who want comparative information. The Alliance does plan to flesh out the community-specific portals to be tailored toward a *C. elegans* researcher, for example, such that the search is focused on *C. elegans* entities, and eventually, the order of content is either configurable as in current WormBase.org or tailored to users with focus on 1 organism, with function being more important than orthology as an example.

## Data Availability

All WormBase and Alliance code and information are freely available. Specifically, WormBase code is freely available at https://github.com/WormBase. WormBase data are available for download and programmatically by API as described at https://wormbase.org/about/userguide/for_developers/api-rest#10–10. Alliance data are at https://www.alliancegenome.org/downloads. Alliance code is available at https://github.com/alliance-genome. Alliance APIs are available at https://www.alliancegenome.org/swagger-ui/.
